# Thwarting resistance: MgrA inhibition with methylophiopogonanone a unveils a new battlefront against *S. aureus*

**DOI:** 10.1038/s41522-024-00485-w

**Published:** 2024-02-27

**Authors:** Xuerui Guo, Li Wang, Jinlong Zhang, Quan Liu, Bingmei Wang, Da Liu, Fei Gao, Gongga Lanzi, Yicheng Zhao, Yan Shi

**Affiliations:** 1 School of Pharmaceutical Science, Jilin University, Changchun, China; 2 Clinical Medical College, Changchun University of Chinese Medicine, Changchun, China; 3 Center for Pathogen Biology and Infectious Diseases, Key Laboratory of Organ Regeneration and Transplantation of the Ministry of Education, The First Hospital of Jilin University, Changchun, China; 4 School of Pharmacy, Changchun University of Chinese Medicine, Changchun, China; 5 Department of Laboratory Animals, College of Animal Sciences, Jilin University, Changchun, Jilin China; 6 Tibet University Medical College, Tibet, China; 7 State Key Laboratory for Diagnosis and Treatment of Severe Zoonotic Infectious Diseases, Key Laboratory for Zoonosis Research of the Ministry of Education, Institute of Zoonosis, and College of Veterinary Medicine, Jilin University, Changchun, China

**Keywords:** Bacteria, Biofilms

## Abstract

Limitations in the clinical treatment of *Staphylococcus aureus* (*S. aureus*) infections have arisen due to the advent of antibiotic-resistant strains. Given the immense potential of therapeutic strategies targeting bacterial virulence, the role of MgrA as a pivotal virulence determinant in *S. aureus*-orchestrating resistance, adherence, and hundreds of virulence targets—becomes indispensable. In this investigation, leveraging advanced virtual screening and fluorescence anisotropy assays, we discerned methylophiopogonanone A (Mo-A), a flavonoid derivative, as a potent disruptor of the MgrA-DNA interaction nexus. Subsequent analysis revealed that Mo-A effectively inhibits the expression of virulence factors such as Hla and Pvl in *S. aureus* and markedly reduces its adhesion capability to fibrinogen. On a cellular landscape, Mo-A exerts a mitigating influence on the deleterious effects inflicted by *S. aureus* USA300 on A549 cells. Furthermore, our data indicate that Mo-A downregulates the transcription of genes associated with immune evasion, such as nucleases (nuc), Staphylococcal Chemotaxis Inhibitory Protein (chips), and Staphylococcal Complement Inhibitor (scin), thereby undermining immune escape and amplifying neutrophil chemotaxis. Upon application in an in vivo setting, Mo-A assumes a protective persona in a murine model of *S. aureus* USA300-induced pneumonia and demonstrates efficacy in the *Galleria mellonella* infection model. Of note, *S. aureus* displayed no swift acquisition of resistance to Mo-A, and the effect was synergistically enhanced when used in combination with vancomycin. Our findings add substantive weight to the expanding field of virulence-targeted therapeutic strategies and set the stage for more comprehensive exploration of Mo-A potential in combating antibiotic-resistant *S. aureus*.

## Introduction

*Staphylococcus aureus* (*S. aureus*), ubiquitously present within the human microbiota, has earned an infamous reputation stemming from its propensity to trigger diverse life-threatening infections alongside its formidable capacity for acquiring multidrug resistance^1^. With its adhesion and invasion abilities, *S. aureus* can hide within human tissues, form biofilms, and secrete an array of virulence factors detrimental to host immunity^2,3^. Notably, *S. aureus* possesses the ability to survive even within phagocytic immune cells, exploiting them as Trojan horses to infiltrate, colonize, and invade various sites within the human body^4^.

*S. aureus* has evolved a multitude of adaptive strategies, enabling this pathogen to surmount physical, immune, and chemical barriers imposed by the host. The Centers for Disease Control and Prevention (CDC) has classified both vancomycin-resistant *Enterococci* (VRE) and methicillin-resistant *Staphylococcus aureus* (MRSA) as grave health threats on a global scale^5^. Considering this, comprehending the pathogenic mechanisms underlying *S. aureus* infections and pioneering innovative preventive methodologies remain paramount imperatives in the field.

Emerging strategies in combatting severe bacterial infections now incorporate pathogen-specific therapeutic approaches such as monoclonal antibodies (mAbs), nanomaterials, bacteriophages, and antivirulence therapy^6,7^. Contrasting the existing antibiotics, medications that target pathogenicity and virulence traits could confer an upper hand to the host immune system with respect to bacterial adaptability and infection control^8^. Additionally, this methodology could introduce novel avenues to diminish or circumvent the evolutionary pressure typically observed. In light of recent advances, scholarly attention has been increasingly gravitating toward the identification and understanding of key virulence factors within *S. aureus*, with the aim of devising innovative, targeted therapeutic strategies^9^. The expression of bacterial virulence constitutes an intricate tapestry of interrelated events, executed with remarkable precision and coordination^10^. This well-choreographed process is governed by an array of regulatory factors, some of which are well documented, while others remain enigmatic. Regulators play a pivotal role in modulating the expression and pathways of various genes, thereby dictating the pathogenic processes of bacteria.

Among numerous virulence regulators, MgrA has emerged as a pivotal global regulator within this pathogen. MgrA, a DNA-binding multifaceted gene regulator, orchestrates the expression of over 350 genes impacting antibiotic resistance, toxin production, and biofilm formation^11,12^. The transcription of the MgrA gene is governed by two promoters, P1 and P2, with the stability of the mRNA transcript originating from the P2 promoter being enhanced by RNAIII. In addition to the action of RNAIII, MgrA biosynthesis is subject to regulation by another small RNA-mediated mechanism^12^. Mutations or inhibition of the *mgrA* gene within *S. aureus* diminish virulence, thus highlighting its promising potential as a target for novel antivirulence therapies^13,14^. Developing MgrA-specific inhibitors could pave the way for novel therapeutic strategies to combat recalcitrant *S. aureus* infections.

Flavonoids, a class of naturally occurring polyphenolic compounds, have drawn increasing attention due to their broad biological activities and potential therapeutic applications^15–17^. The structural diversity of flavonoids allows them to interact with multiple targets within bacterial cells, offering strategic approaches for therapeutic MRSA^18,19^. Additionally, the low toxicity and high tolerance of flavonoids within the human body render them appealing candidates for the development of antivirulence agents.

In our research, we embarked on a virtual screening process, employing libraries of flavonoid, alkaloid, and terpenoid compounds. To our delight, flavonoids indeed stood head and shoulders above the rest in this screening. Subsequently, from the category of flavonoids, we identified methylophiopogonanone A (Mo-A) as a molecule capable of disrupting the biological function of MgrA. The current study aims to elucidate the underlying mechanism by which Mo-A exerts inhibitory effects on MgrA, offering a novel candidate compound for combating multidrug-resistant *S. aureus* infections.

## Results

### Mo-A as an inhibitor of mgrA in S. aureus

**Fig. 1:**
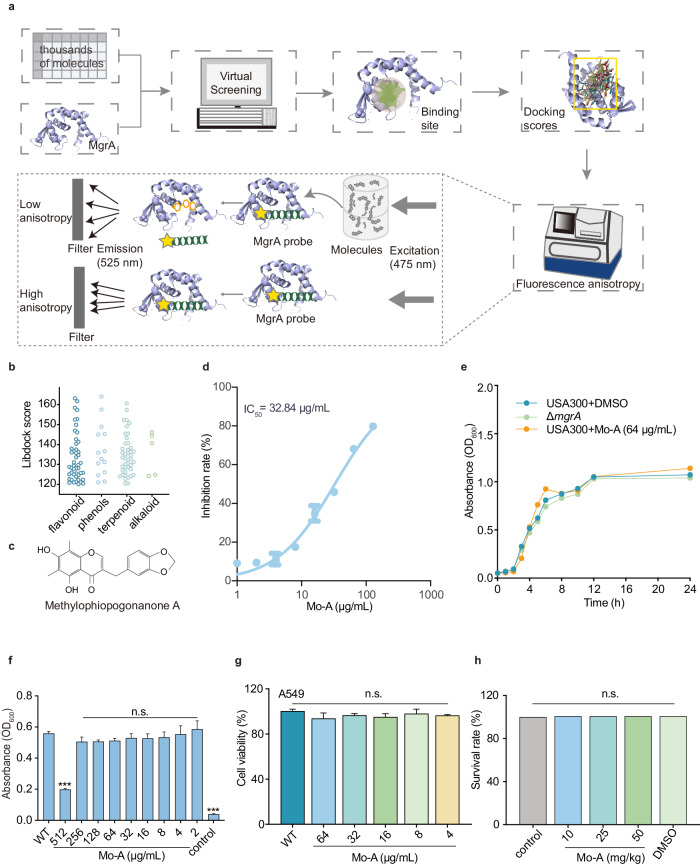
The inhibitory effect of Mo-A on MgrA activity. **a** Virtual screening and fluorescence anisotropy were used to screen MgrA inhibitors from natural small molecule compounds. **b** Natural small molecule compounds with LibDock absolute scores greater than 120 were classified. **c** The chemical structure of Mo-A. **d** The effect of Mo-A on MgrA activity with an IC_50_ of 32.84 μg/mL (95.91 μM). **e** Growth curves of *S. aureus* USA300 with or without Mo-A treatment (64 μg/mL). **f** The MIC of Mo-A against *S. aureus* USA300 (WT group). **g** The viability of A549 cells measured by MTT assay after exposure to various concentrations of Mo-A (0 to 64 μg/mL). **h** Assessment of the in vivo toxicity of Mo-A in *Galleria mellonella*. *P < 0.05, **P < 0.01, ***P < 0.001 compared to the WT group. Error bars represent standard error of the mean.

In preliminary investigations and through the synthesis of extant studies, it was determined that alkaloids, terpenes, and flavonoids manifest a pronounced propensity for the abrogation of virulence in *S. aureus*. This revelation precipitated the selection of small-molecule libraries encompassing these triumvirates of compounds, which were subjected to virtual screening via Discovery Studio (Supplementary Figure 1). The selection process is depicted in Fig. 1a. Intriguingly, within the milieu of compounds boasting a LibDock absolute score in excess of 120, the number of flavonoids emerged as preponderant (Dataset 1 and Fig. 1b). In contrast to the salient role natural products may occupy in fortifying defenses against bacterial pathogens, the CDOCKER approach employing virtual screening was amalgamated with an assemblage of 79 small molecules curated from our laboratory for more incisive scrutiny. Of the CDOCKER scores obtained, eight exhibited absolute values greater than 100 (Dataset 2). These thresholds were established to ensure a high likelihood of significant binding while maintaining a manageable number of lead compounds for further evaluation^20^.

Subsequently, based on the eight candidate compounds identified through two rounds of virtual screening, we assessed their inhibitory effects on MgrA using the fluorescence anisotropy method. Among them, Mo-A demonstrated notably high inhibitory efficiency, with a calculated IC_50_ of 32.84 μg/mL, and was thus regarded as a candidate compound for further investigation (Fig. 1c, d and Supplementary Fig. 2).

### Mo-A does not affect the growth of S. aureus and is not significantly toxic

Distinct from the bactericidal action of antibiotics, antivirulence drugs aim to attenuate the production of virulence factors by bacteria without directly impeding their growth. Accordingly, we investigated whether Mo-A, at varying concentrations, influences the growth kinetics of *S. aureus* USA300 by monitoring its growth over time. As depicted in Fig. 1e, there was no discernible difference in the growth rate between the 64 μg/mL Mo-A treatment group and the DMSO control group. This implies that Mo-A does not impede the growth of *S. aureus* USA300 at this concentration, aligning with the anticipated characteristics of an antivirulence drug. Additionally, we determined the minimum inhibitory concentration (MIC) of Mo-A against USA300 to be 512 μg/mL using the microdilution method (Fig. 1f). Furthermore, cellular toxicity experiments revealed that Mo-A, even at concentrations significantly exceeding its IC_50_, does not adversely affect the survival of A549 cells, attesting to its safety profile (Fig. 1g). Subsequently, we extended our evaluation of Mo-A toxicity in vivo using final instar larvae of *Galleria mellonella*. The larvae were administered 10, 25 or 50 mg/kg Mo-A via injection into the last right leg hole. Their status and degree of melanization were assessed daily, with survival tracked over a span of 5 days posttreatment. Our results demonstrated that both 10, 25 and 50 mg/kg doses of Mo-A were well tolerated, with no instances of mortality observed in the *Galleria mellonella* larvae (Fig. 1h). These findings provide a robust foundation for further exploration of Mo-A as a potential antivirulence drug.

### In vitro suppression of staphylococcal hemolytic activity and Hla expression by Mo-A

**Fig. 2:**
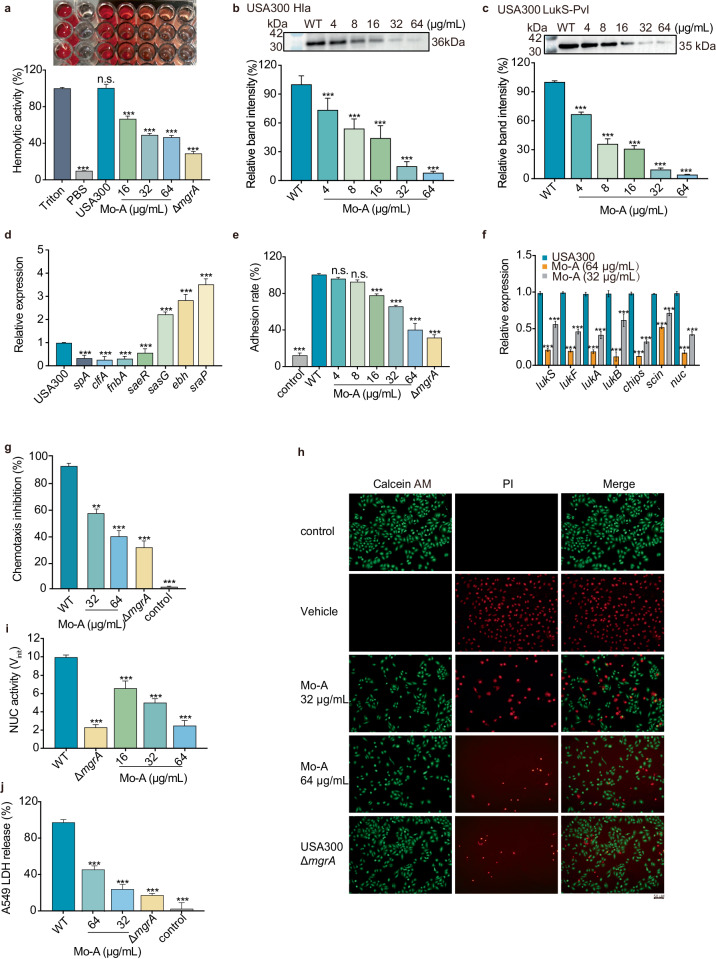
Influence of Mo-A on the virulence factors regulated by MgrA. **a** The effects of Mo-A on the hemolytic activity of *S. aureus* USA300 were evaluated. A reduction in the number of erythrocytes lysed by hemolysin was observed, with the hemolytic capability inversely proportional to the Mo-A concentration, demonstrating a dose-dependent relationship. Triton group: Triton X-100 group. **b** Mo-A was found to inhibit the expression of hemolysin in *S. aureus* USA300. Immunoblot images have been cropped for illustrative purposes. **c** Western blot analyses examined the impact of Mo-A on the expression of Pvl in *S. aureus* USA300. Immunoblot images have been cropped for illustrative purposes. **d** Changes in the transcription levels of virulence genes in *S. aureus* at different concentrations of Mo-A were studied using qPCR. **e** Crystal violet staining was employed to visualize and quantify the effects of Mo-A on biofilm formation *in S. aureus*. **f** Relative expression of MRSA genes after treatment with various concentrations of Mo-A (*n* = 3 per group). **g** The ability of *S. aureus* culture supernatants to block neutrophil chemotaxis was measured. **h** The effect of Mo-A on *S. aureus* infection in A549 cells was examined using live/dead cell detection (Scale bar: 50 μm). **i** Nuclease activity in culture supernatants was measured. **j** A549 cells LDH analysis. *P < 0.05, **P < 0.01, ***P < 0.001 compared to USA300 (WT group). The control group was the blank group. Error bars represent standard error of the mean.

Hemolysin alpha (Hla), a virulence target regulated by *S. aureus* MgrA, is primarily secreted during the log phase of growth. It induces changes in the transmembrane ion gradient of host cells, disrupting membrane integrity and leading to the death of various cell types. To assess the effect of Mo-A on the Hla of *S. aureus*, hemolysis experiments were further conducted. Mo-A inhibited the Hla activity of both *S. aureus* USA300 and Newman in a dose-dependent manner, while Mo-A itself was not hemolytic (Fig. 2a, Supplementary Figs. 3 and 4a). When the concentration of Mo-A was 64 μg/mL, the hemolytic ability of the *S. aureus* supernatant was significantly inhibited, reaching only 8.08 ± 1.60%. Similar effects were observed in *S. aureus* Newman, with Mo-A dose-dependently inhibiting its hemolytic activity. At a concentration of 64 μg/mL, the hemolytic potential of the *S. aureus* supernatant was significantly suppressed by Mo-A (Fig. 2b, Supplementary Figs. 4b and 5c).

### In vitro suppression of Staphylococcal panton-valentine leukocidin (Pvl) expression by Mo-A

MgrA regulates a diverse repertoire of virulence factors and toxins, encompassing Hla and leukocidins, that profoundly influence the pathogenesis of infection and subversion of host immune responses^21^. Of particular note are the single-component and two-component pore-forming toxins, including Hla and Pvl, which selectively target immune cell membranes, causing membrane disruption as a striking hallmark of their mode of action. As depicted in Fig. 2c, Supplementary Figs. 4c and 5b, Mo-A demonstrated a dose-dependent inhibition of Pvl activity in both *S. aureus* USA300 and Newman strains. Subsequent western blot results revealed a dose-dependent inhibition of Pvl expression in both *S. aureus* USA300 and Newman strains by Mo-A.

### In vitro suppression of transcription of MgrA-associated virulence factors by Mo-A in S. aureus

In addition to regulating Hla, studies have shown that MgrA can form a regulatory cascade with ArlRs, which is closely associated with intravascular adhesion and colony aggregation, influencing coagulase, certain surface proteins, and exogenous proteins^22^. We subsequently evaluated the impact of Mo-A on the transcription levels of genes that play a crucial role during *S. aureus* infection (*sasG*, *clfA*, *ebh*, *sraP*, *spA*, *fnbA*, *saeR*) through qPCR. As shown in Fig. 2d, compared with the untreated group, 64 μg/mL Mo-A reduced the transcription levels of *spA*, *clfA*, *fnbA* and *saeR* virulence-associated factors by 3-fold, 3.9-fold, 3.2-fold, and 1.8-fold, respectively. In contrast, the transcription of genes encoding the surface proteins *saeG*, *ebh* and *sraP* increased by 2.2-fold, 2.8-fold and 3.5-fold, respectively. Replacing different housekeeping genes *rplD*, the trends of upregulation and downregulation of genes regulated by *mgrA* are essentially similar (Supplementary Fig. 6a). These results indicate that Mo-A can suppress the transcript levels of MgrA-related regulatory genes in vitro.

### Mo-A suppresses the adhesive capacity of S. aureus to fibrinogen

The capacity of *S. aureus* for robust host infection is attributed to the diverse gene product expression intrinsic to the organism^23^. Its pervasive dissemination and subsequent infection within hospital settings is intimately linked with its proficiency in adhesion. We exploited crystal violet staining to assess whether Mo-A could intervene in the adhesion of *S. aureus* to fibrinogen. As depicted in Fig. 2e, a phenomenon exhibited dose dependency with increasing concentration. At a concentration of 64 μg/mL, Mo-A notably attenuated the adhesion rate of *S. aureus* to fibrinogen to 40.31 ± 6.93%.

### Mo-A modulates immune evasion by staphylococcus aureus and enhances neutrophil chemotaxis

*S. aureus* secretes an array of immune modulatory factors, such as leukocidins, chips, scin, and nucleases (nuc), which empower its ability to both impair neutrophil function and induce neutrophil lysis, thereby evading host immunity. Through qPCR analyses, we observed a significant reduction in the secretion of leukocidins, chips, scin, and nuc in response to increasing concentrations of Mo-A (Fig. 2f and Supplementary Fig. 6b). The supernatant from MRSA notably curtails chemotaxis. However, this suppressive effect was diminished with incremental Mo-A concentrations, resulting in a performance less effective than that of MRSA (Fig. 2g). Concurrently, increasing dosages of Mo-A induced a dose-correlated decline in supernatant nuclease activity (Fig. 2i).

### Mo-A attenuates S. aureus-induced damage in A549 cells

By demonstrating that Mo-A can mitigate the pathogenic effects of MgrA, we further evaluated the protective effects of Mo-A on A549 cells infected with *S. aureus*. Following infection, A549 cells were administered graded doses of Mo-A and subsequently dual-stained using calcein AM/PI. Microscopic evaluation under fluorescence illumination revealed a pronounced green fluorescence signal juxtaposed with scant red fluorescence (Fig. 2h). This observation underlines the remedial potential of Mo-A in mitigating the destructive effects incited by *S. aureus* within A549 cells, particularly at a concentration of 64 μg/mL. This inference is further supported by the lactate dehydrogenase (LDH) release assay, which showed a marked decrease in LDH extrusion from the jeopardized A549 cells after Mo-A administration (Fig. 2j).

### Direct interactions between Mo-A and MgrA

**Fig. 3:**
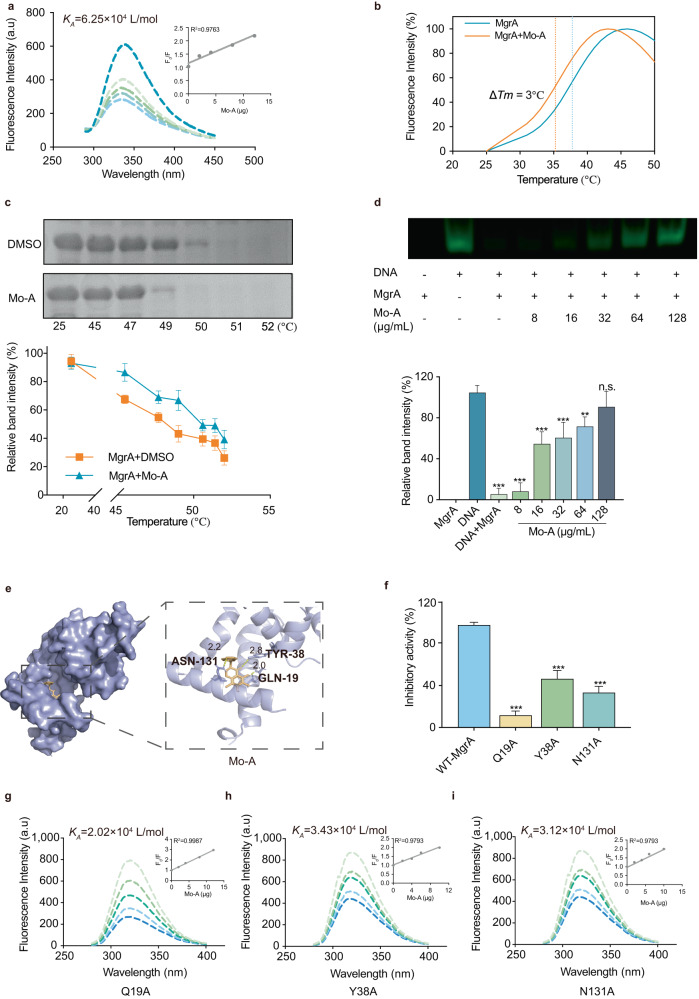
Establishing the direct interaction between Mo-A and MgrA. **a** Fluorescence quenching was employed to gauge the affinity between Mo-A and MgrA. **b**, **c** The influence of Mo-A on the thermal stability of MgrA was ascertained through a thermal shift assay (TSA) and a cellular thermal shift assay (CETSA). SDS-PAGE gel has been cropped for illustrative purposes. **d** Mo-A dose-dependently inhibited the interaction between MgrA and the hla promoter and the analysis of relative band intensity. EMSA gel has been cropped for illustrative purposes. **e** Molecular docking simulated the key amino acid sites of interaction between Mo-A and MgrA. **f** The effect of Mo-A at a concentration of 64 μg/mL on the activity of MgrA mutants was explored by fluorescence anisotropy. The effect of Mo-A at a concentration of 64 μg/mL on the activity of MgrA mutants of Q19A **g**, Y38A **h**, and N131A mutants **i** were explored by fluorescence quenching. *P < 0.05, **P < 0.01, ***P < 0.001 compared to USA300 (WT group). Error bars represent the standard error of the mean.

Subsequently, we analyzed the binding kinetics between Mo-A and MgrA through fluorescence quenching. With the increase in Mo-A concentration, the fluorescence intensity of MgrA decreased continuously, forming static quenching. The calculated binding constant *K*_*A*_ was 6.25×10^4^ L/mol (Fig. 3a). The binding between proteins and small molecules can lead to tighter (or looser) protein structures, consequently changing the melting temperature (Tm). By taking advantage of the ability of SYBO fluorescent dyes to bind with the hydrophobic area of unfolded proteins and emit fluorescence signals^24,25^, we assessed the interaction between Mo-A and MgrA through a thermal shift assay (TSA). Our results demonstrated that the Tm of the MgrA protein was 38°C, whereas after combining with Mo-A, it decreased to 35°C without the influence of fluorophore and Mo-A (Fig. 3b and Supplementary Fig. 7). This marked difference in Tm, designated ∆Tm, surpasses 3 °C, thereby inferring a direct interaction between Mo-A and MgrA.

Furthermore, we conducted a cellular thermal shift assay (CETSA) using *E. coli* lysate carrying pet28a::mgrA. As the temperature increased, a significant difference was observed between the Mo-A-treated group and the DMSO-treated group (*P* > 0.001), which suggested a direct interaction between Mo-A and MgrA (Fig. 3c). These findings pointed toward a strong interaction between Mo-A and MgrA. Concurrently, evidence from the electrophoretic mobility shift assay (EMSA) authenticated that Mo-A imposes a dose-dependent inhibition on the binding capacity of MgrA to the hla promoter, thus substantiating its regulatory function (Fig. 3d).

To further elucidate the key amino acid residues involved in the binding of Mo-A and MgrA, we performed molecular docking (Fig. 3e and Supplementary Table 2). The docking analysis confirmed the interaction between Mo-A and MgrA, supporting its potential as a promising candidate for further investigation^14^. Residues GLN-19, TYR-38, and ASN-131 were identified as the key amino acids for Mo-A and MgrA binding. Based on the MMGBSA method, the binding free energy *∆G*_*bind*_ of the MgrA-Mo-A complex was calculated to be -6.7 kcal/mol (Supplementary Table 3). Next, we mutated these key amino acid residues and performed fluorescence anisotropy and fluorescence quenching analysis. The results showed that the inhibitory activity of Mo-A with mutated GLN-19, TYR-38, and ASN-131 sites was lower than that with unmutated proteins, validating that the binding of Mo-A and MgrA (Fig. 3f). As the concentration of Mo-A increased, there was a continuous decline in the fluorescence intensity observed in MgrA mutants. The binding constant (*K*_*A*_) of GLN-19, as calculated, showed the lowest value (Fig. 3g–i). These findings pointed toward a strong interaction between Mo-A and MgrA.

### Mo-A protects Galleria mellonella from S. aureus infection and reduces melanization

**Fig. 4:**
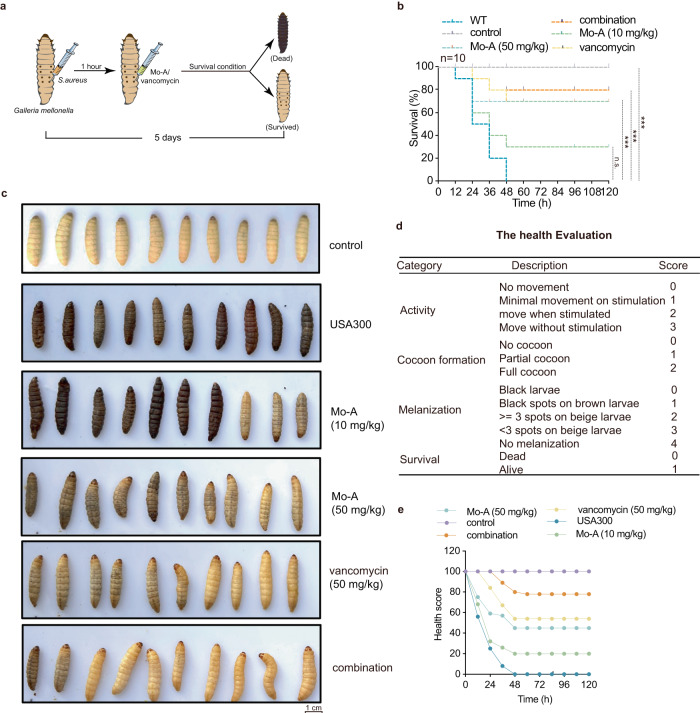
Mo-A alleviates the pathogenic effect of *S.aureus* on *Galleria mellonella*. **a** Schematic representation of *S. aureus* USA300 infection and Mo-A with/without vancomycin treatment regimen in *Galleria mellonella*. **b** Survival outcome of *Galleria mellonella* larvae (*n* = 10) postinfection with a lethal dose of *S. aureus* USA300 following treatment interventions with Mo-A, vancomycin, Δ*mgrA*, and their respective combinations. **c** Representative photographic illustrations of various *Galleria mellonella* larval groups in which scale bars were 1 cm (*n* = 10). **d** Health Index Scoring System for *Galleria mellonella*. **e** Based on this scoring paradigm, the derived scores for each group were indicative of a significant ameliorative effect conferred by Mo-A and its synergistic action when combined with vancomycin.

As an insect with high similarity to vertebrates in terms of its immune system, *Galleria mellonella* serves as an excellent model for drug efficacy validation. Through monitoring its activity, cocoon formation, melanization, and survival status, we assessed the therapeutic effects of drugs. To this end, we performed a 5-day survival observation after injecting the *Galleria mellonella* larvae with USA300 inoculum and administering the test drugs (Fig. 4a).

Over time, we observed that the survival rate of the *Galleria mellonella* larvae gradually increased with increasing concentrations of Mo-A, indicating a substantial attenuation of MRSA infection. Eventually, the survival rate reached 70% (Fig. 4b). Additionally, on the fifth day, we captured images showing a significant improvement in survival rates with combination antibiotic treatment (Fig. 4c). Considering the health assessment scores, we firmly believe in the potent efficacy of Mo-A combined with antibiotics in reducing mortality caused by MRSA infection in the *Galleria mellonella* model (Fig. 4d, e).

### Mo-A protects mice from S. aureus pneumonia infection and reduces lung inflammation

**Fig. 5:**
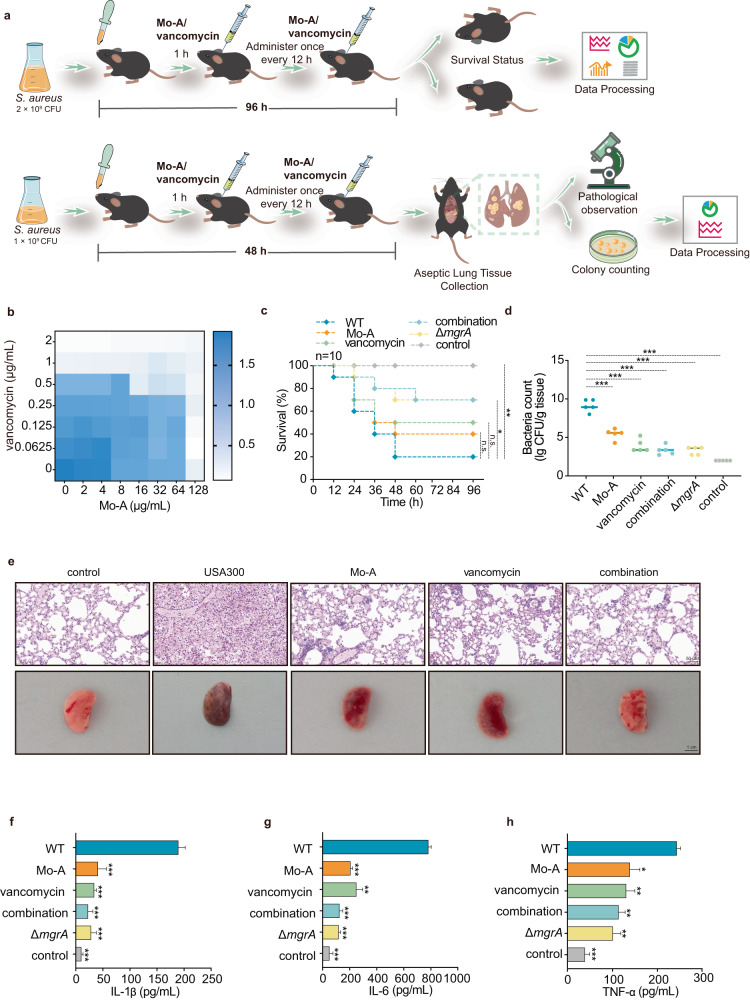
Therapeutic effects of Mo-A and vancomycin against *S. aureus* USA300-induced pneumonia in mice. **a** Experimental model of MRSA-induced pneumonia in C57BL/6 J mice. **b** Checkerboard assay demonstrating the combined effects of Mo-A and vancomycin against *S. aureus* USA300. **c** The impact on survival rates of C57BL/6 J mice (*n* = 10) infected with a lethal dose of *S. aureus* USA300 treated with Δ*mgrA*, Mo-A, vancomycin, and their combination. **d** Mice were euthanized after 48 h, and lung tissue samples were collected to determine CFU per gram (*n* = 5). The results are presented as a scatter plot and median. **e** Lungs were grossly evaluated for all mice and histopathology of lung tissue from each group of mice (H&E staining). Scale bar of histopathology: 1 cm and scale bar of gross analysis: 50 μm. **f**–**h** Levels of the proinflammatory cytokines IL-1β, IL-6, and TNF-α in bronchoalveolar lavage fluid (*n* = 3). Data are presented as the mean ± standard deviation. The control group was treated with PBS group. *P < 0.05, **P < 0.01, ***P < 0.001 compared to USA300 (WT group).

Given the association of *S. aureus* USA300 with severe respiratory infections, we sought to determine the therapeutic potential of Mo-A against pneumonia induced by *S. aureus* USA300 (Fig. 5a). To elucidate a potential synergistic action between Mo-A and vancomycin (the antibiotic of choice against MRSA), a checkerboard assay was performed. By determining the MIC values of antibiotics alone against *S. aureus* USA300 and then combining them with 64 μg/mL Mo-A, we found that vancomycin had the lowest fractional inhibitory concentration index (FICI) value (Supplementary Table 4). An FICI of 0.375 further substantiates the existence of a synergistic interaction between 16 μg/mL Mo-A and 1/4 MIC vancomycin (Fig. 5b). When 1/4 MIC of vancomycin was used in combination with 16 μg/mL of Mo-A, it effectively inhibited the number of USA300 bacteria during both the exponential growth (Supplementary Fig. 8).

In determining the final dosage of the mortality study, we integrated previous research with the findings from our preliminary experiments, which are presented in Supplementary Fig. 9^20,26^. Without Mo-A treatment, the survival rate was merely 20% within 96 h post-infection; however, treatment with a combination of Mo-A and vancomycin markedly increased this rate to 70% (Fig. 5c). These data suggest that Mo-A significantly enhances the survival rate of *S. aureus* pneumonia mice. Subsequently, we quantified the bacterial load in the lung tissues of mice from each group. After challenge with 1 × 10^9^ CFU of *S. aureus*, treatment with the combination of Mo-A led to a notable decrease in the recovered bacterial count in lung homogenates, reducing from 9.20 ± 0.84 lg CFUs/g to 5.24 ± 0.74 lg CFUs/g (*P* < 0.001). However, the combination revealed a distinctly superior outcome of 2.96 ± 0.66 lg CFUs/g (Fig. 5d).

Additionally, to assess the extent of lung damage in mice, lung tissues were collected. The lungs from infected mice presented a dark red color and noticeable swelling, while lungs from Mo-A-treated mice appeared pinkish, showing significant alleviation. To better evaluate the pathological changes in the lungs of mice infected with *S. aureus* due to Mo-A treatment, we conducted H&E staining. At the early stage of infection, *S. aureus* releases a large amount of toxins and other exoproteins, leading to substantial immune cell infiltration into the lungs. Under microscopy, we observed prominent inflammatory cell infiltration in the lungs of the infection group, while combination treatment led to a notable decrease in inflammatory cell accumulation in the alveolar space, and the alveolar structure became more distinct (Fig. 5e). We further examined changes in the levels of several typical inflammatory factors in the bronchoalveolar lavage fluid (BALF) of each group. IL-1β, IL-6, and TNF-α levels in the BALF of Mo-A-treated infected mice all showed varying degrees of decrease (Fig. 5f–h). These results suggest that combination treatment can effectively improve the survival rates of *S. aureus*-infected mice, reduce the lung bacterial load, and alleviate lung damage.

## Discussion

Antibiotics have been considered foundational in the fight against bacterial infections. However, the efficacy of these critical pharmaceutical agents is increasingly being compromised due to the relentless development of resistance by bacterial populations. Over the past 15 years, there has been a consistent and substantial increase in the utilization of antibiotics, a trend further exacerbated by the prolonged SARS-CoV-2 pandemic. Notably, a significant majority of patients suffering from severe COVID-19 have been prescribed antibiotics as a prophylactic measure to preempt potential secondary bacterial infections^27,28^. Antivirulence strategies endorsed by the FDA with therapies such as raxibacumab in trials could reduce bacterial resistance and preserve host microbiome equilibrium^29,30^. Among these, *S. aureus* demands paramount attention. Considering the indomitable resistance that *S. aureus* has manifested against a panoply of antibiotics, the deployment of solitary antibiotic agents often falters in achieving coveted therapeutic outcomes. Extensive research underscores MgrA as a pivotal global regulator of virulence determinants, exerting influence over numerous virulence targets^31^. Targeting MgrA emerges as a plausible strategy to combat multidrug-resistant *S. aureus*.

In recent years, there has been an emerging recognition of the formidable therapeutic potential harbored within plant-derived compounds, particularly in combating an array of infectious diseases^32,33^. The remarkable diversity of secondary metabolites present in plants, including alkaloids and polyphenols, exhibits a wide spectrum of biological activities that equip them with the ability to tackle various ailments^34^. Plant-derived small molecules, often with a molecular weight below 500, are commonly referred to as phytoalexins or phytochemicals. Although their antimicrobial activities may be more subtle compared to conventional antibiotics, they offer a diverse arsenal of mechanisms targeting microbes, which turns out to be a valuable asset^35^.

Notably, these natural compounds often synergize with established antibiotics, potentiating their antimicrobial effects^36,37^. This synergy heralds an efficacious strategy to address the escalating issue of antibiotic resistance, as it diminishes the likelihood of pathogenic adaptation and resistance development. Virtual screening revealed that Mo-A, as one of flavonoids stand out among the natural molecular library for their strong interaction potential with MgrA. Mo-A is heralded for its antioxidative and anti-inflammatory characteristics^38^. Earlier scholarly endeavors have indicated that a traditional medicine concoction comprising Ophiopogon japonicus (Mai Dong in Traditional Chinese Medicine) might be instrumental in conferring cerebral fortification against the scourge of I/R injury^39^.

In the present study, utilizing the fluorescence anisotropy technique, we conducted a systematic screening for MgrA inhibitors, leveraging the intrinsic ability of MgrA to selectively bind to specific DNA sequences. Mo-A was identified as an MgrA inhibitor. We further expanded the therapeutic potential of Mo-A, demonstrating its utility in the realm of anti-infective applications.

Safety remains a paramount concern in drug discovery and development. Therefore, in parallel to our mechanistic investigations, we assessed the safety profile of Mo-A both at the cellular level and in a more complex biological context, specifically the *Galleria mellonella* larvae. *Galleria mellonella* larvae, capable of surviving across a wide temperature range needed for virulence factor expression, have proven to be a remarkably sturdy model for evaluating drug toxicity^40^. Despite their lack of adaptive immune responses, these organisms display innate immune responses that strikingly mirror those seen in vertebrates^41^, making them an advantageous model for evaluating potential drug effects in biologically relevant environments. Our assessments verified that Mo-A, at concentrations needed for the inhibition of MgrA, did not negatively impact the survival of either A549 cells or *Galleria mellonella* larvae.

Patients with *S. aureus* infections often have comorbidities and take multiple drugs, making it essential to understand Mo-A’s effects on P450 enzymes. As a potential dual inhibitor of P450 enzymes, Mo-A exhibits reversible inhibition on certain P450 enzymes (such as CYP1A, 2C8, 2C9, 2C19, and 3 A) and irreversible inhibition on CYP2D6 and CYP2E1. These actions of Mo-A might be beneficial, such as reducing the active metabolites of certain drugs or toxins, thereby lowering the risk of specific cancers and alcohol-induced liver damage. Mo-A’s irreversible inhibition of CYP2E1 could help decrease the active metabolism of drugs like acetaminophen, reducing potential hepatotoxicity. Meanwhile, Mo-A’s inactivation of CYP2D6 might impact the metabolism of certain medications commonly used by elderly patients, such as antiarrhythmic drugs (e.g., encainide), antihypertensives (e.g., nicergoline), and antidepressants (e.g., clomipramine), potentially increasing their plasma concentrations, especially those with narrow therapeutic indices^42^. Therefore, clinical attention is needed for potential interactions between Mo-A and these drugs to prevent adverse reactions.

**Fig. 6:**
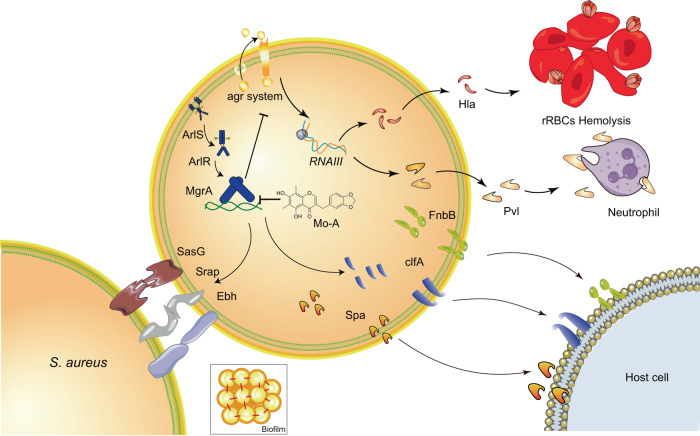
Disruption of MgrA-mediated virulence factors by Mo-A intervention. Regulation of MgrA by the ArlS-ArlR two-component system. Mo-A-mediated perturbation impedes MgrA-DNA interactions, compromising its regulatory efficacy over the downstream Agr quorum sensing circuitry. This interference manifests as reduced biogenesis of the Hla heptameric complex, curtailing erythrocytic hemolysis. Concurrently, a diminished release of the cytotoxin Pvl is evident, suggesting attenuated immune evasion tactics. Notably, Mo-A also modulates MgrA-dependent bacterial adhesiveness and biofilm morphogenesis.

The two-component ArlRS system, in conjunction with the global regulator MgrA in *S. aureus*^43^, is recognized to shape a crucial regulatory cascade that plays an instrumental role in various physiological processes, including intravascular adhesion and colony aggregation (Fig. 6). Of note is MgrA, as it acts as a central switch in the aggregation mechanism by predominantly repressing the expression of surface proteins^44,45^. In line with our anticipations, Mo-A profoundly downregulated the transcript levels of *clfA*, *spA*, and *fnbA*, thereby inhibiting *S. aureus* adhesion to fibrinogen in a dose-responsive manner. Additionally, with Mo-A present, we documented an augmented transcriptional activity of genes encoding pivotal surface proteins, namely, *ebh*, *sraP*, and *sasG*. The enhanced expression of these large surface proteins introduced spatial constraints, culminating in diminished bacterial adhesion to the host.

Beyond its impact on surface proteins, Mo-A also critically modulates the expression of potent virulence factors in *S. aureus*, namely, Hla and Pvl. Hla acts by disrupting host cell membranes, triggering cell lysis and subsequent tissue damage. Conversely, Pvl specifically targets leukocytes, compromising the immune responses of the host and escalating the severity of infections. The presence of Mo-A significantly reduced the pathogenicity of *S. aureus*.

In the context of infections, the formation of neutrophil extracellular traps (NETs) is a pivotal host defense mechanism specifically tailored to combat pathogens^46^. However, pathogens such as *S. aureus* have evolved sophisticated strategies to counteract these defenses. Notably, the bacterium produces nucleases capable of degrading NETs, thus allowing it to elude phagocytosis within abscesses and proliferate at the infection site^47^. The bacterium employs a strategic mechanism, utilizing an array of immune-evasive molecules, to hinder the migration of neutrophils to sites of infection. Critical among these molecules are bicomponent leukotoxins, which disrupt the membrane integrity of phagocytes, chemotactic inhibitors that misdirect neutrophil navigation, complement inhibitor chips that interfere with opsonization, and staphylococcal complement inhibitor (SCIN), which impedes complement cascade activation. Our study has elucidated the involvement of these factors in bacterial immune evasion tactics. These elements are vital for the ability of bacterium to evade the immune system and present promising targets for therapeutic interventions, potentially improving clinical treatments. Furthermore, these molecules are regulated by the MgrA system^48,49^. Subsequent qPCR analyses robustly affirmed a marked decline in the production of two leukotoxins (*lukSF* and *lukAB*) and observed diminished expression of *chips* and *scin*, corroborating our preliminary hypotheses.

When developing new therapeutic compounds, it is essential to remain cognizant of panassay interference compounds (PAINS), notorious for high false-positive rates and interference with screening methodologies, often leading to stalling in drug development^50^. A rigorous inspection of the database (http://www.swissadme.ch/index.php) confirmed that Mo-A is devoid of PAINS traits (Dataset 3). Our subsequent thermal stability experiments further substantiated the non-PAINS nature of Mo-A, as we observed that Mo-A modulates the thermal stability of MgrA via direct conjugation. The significant affinity between Mo-A and MgrA was further confirmed by fluorescence quenching. Further molecular docking studies provided insight into the precise interaction pattern between MgrA and Mo-A, with binding site mutations affirming that GLN-19, TYR-38, and ASN-131 serve as the crucial amino acids for binding.

In vivo analysis unequivocally demonstrated that compared with vancomycin group, the combination of Mo-A and vancomycin exhibited no significant difference in bacterial counts, while the combination of Mo-A and vancomycin exerted a substantial protective effect against USA300-induced lethal pneumonia, as substantiated by improved survival rates, dampened pathological hallmarks, and moderated proinflammatory cytokine responses. This finding aligns with our proposed mechanism of action, in which the drug reduces bacterial numbers not by being bactericidal but by decreasing virulence and pathogenicity, which may reduce the likelihood of resistant bacteria.

In conclusion, our study highlights the potent role of Mo-A in modulating MgrA, showcasing its protective effects against MRSA-induced lethal respiratory infections in mice. This discovery positions Mo-A as an exciting prospect for advancing the development of new antimicrobial treatments.

## Methods

### Bacterial strain and growth conditions

*S. aureus* ATCC^®^ BAA-1717™ (USA300-HOU-MR) and ATCC^®^ 25904™ (Newman) were incubated at 37 °C in an aerated environment using tryptic soy broth (TSB) with the addition of antibiotics as needed. *Escherichia coli* was cultured in Luria–Bertani (LB) medium provided by Oxoid. Antibiotic concentrations were carefully selected: for *E. coli*, kanamycin was added at a concentration of 50 μg/mL.

### Construction, expression, and purification of MgrA protein

For recombinant 6-His tagged MgrA protein generation, the pET28a::MgrA expression vector was constructed by amplifying the MgrA gene from the *S. aureus* USA300 genome using PCR with specific primers. After double digestion with *BamH*I and *Xho*I, the PCR product was ligated into the pET28a vector using T4 DNA ligase. The resulting recombinant plasmid was transformed into *E. coli* DH5α for verification and subsequently into *E. coli* BL21 (DE3) for expression. The bacterial culture was grown to an absorbance of 0.8–1.0 at 600 nm, induced with 0.5 mM IPTG, and incubated at 16 °C. Bacteria were lysed using ultrasonication, and the MgrA protein was purified with a HIS-Selected Nickel Affinity Gel system.

### Virtual screening using Discovery Studio

The three-dimensional (3D) structure of the MgrA protein (PDB ID: 1T2P) was acquired from the Protein Data Bank (www.rcsb.org). For virtual screening, the small molecule library was assembled by downloading data from the ZINC website (https://zinc.docking.org/) and incorporating an in-house small molecule in which Compound CIDs were obtained from PubChem (https://pubchem.ncbi.nlm.nih.gov/). The binding sites were predicted based on the shape of the internal receptor within the Discovery Studio^51^. CDOCKER and LibDock, CHARMm-based docking tool applications employing a rigid receptor protocol, were utilized for docking^52,53^. To elaborate, an ensemble of ligand conformations was initially generated using high-temperature molecular dynamics with varied random seeds, considering a desirable number of low-energy orientations. The ligands within the rigid receptor were subjected to final minimization using nonsoftened potentials. For each resulting pose, CHARMM energies and separate interaction energies were calculated^54^. The poses were ranked based on CHARMM energy, and those with the highest scores were retained. The small molecules targeting MgrA were then ranked based on these scores.

### Screening of MgrA inhibitors

The MgrA protein (700 nM, 30 µL) and the compound to be tested (64 µg/mL) were added to a black polystyrene 96-well plate in a buffer solution (10 mM Tris [pH 7.4] and 25 mM sodium chloride) and incubated at room temperature for 30 min. Subsequently, 2 µL of DNA probe (50 nM) was added to each well, and the mixture was incubated for another 20 min. Fluorescence anisotropy values were measured using a microplate reader (475 nm/525 nm). A control group was set up concurrently, where no compound was added. All measurements were performed in triplicate. The inhibitory rate of the drugs was calculated based on the results, and compounds with an inhibitory rate of more than 60% were selected as candidate compounds.

### Minimum inhibitory concentration

Employing the broth microdilution method, as outlined in previous literature, the MIC of Mo-A against *S. aureus* USA300 was determined. A 1:1000 inoculation of the bacterial culture was introduced into fresh CAMHB medium and cultivated until it reached the logarithmic phase. In a 96-well plate, a mixture of 100 μL of CAMHB, *S. aureus* USA300 (1 × 10^5^ CFU) as the WT group, and a series of Mo-A concentrations was prepared, with a medium-only blank control (control group) included. Each concentration was tested in triplicate. After a 16-h incubation period at 37 °C, the MIC was identified as the lowest drug concentration at which bacterial growth was not visible to the naked eye.

### Growth curves

Growth curves were utilized to evaluate the impact of Mo-A on the growth of USA300. *S. aureus* USA300 was resuspended in TSB solution at a 1:100 ratio and incubated overnight. Subsequently, the *S. aureus* culture was adjusted to an OD_600_ of 0.1, followed by the addition of 64 μg/mL Mo-A or an equivalent volume of DMSO. Over the course of the next 24 h, at appropriate time intervals, the OD_600_ values were measured using a microplate reader, and the growth curve of *S. aureus* USA300 was plotted accordingly. All measurements were performed in triplicate.

### MTT

The MTT assay was employed to evaluate the impact of Mo-A on the viability of A549 cells. A549 cells were seeded at a density of 2 × 10^4^ cells per well in a 96-well plate and incubated in a cell culture chamber set at 37 °C with 5% CO_2_, allowing for adherence over a 24 h period. Subsequently, the cells were treated with various concentrations of Mo-A. After a further 24-h incubation period, the culture medium was removed, and each well was supplemented with 5 mg/mL MTT solution, followed by a 4-hour incubation period. Thereafter, 100 μL of DMSO was added to each well to dissolve the formazan crystals, and the absorbance was measured at a wavelength of 490 nm. All measurements were performed in triplicate.

### Hemolytic activity of the Mo-A assay

The defibrinated rabbit red blood was centrifuged at 4 °C and 4000 rpm for 3 min, the supernatant was discarded, and the step was repeated until the supernatant was clear and colorless. Then, 0.5 mL of PBS was added to each tube to resuspend the red blood cells. A negative control using PBS and a positive control with the addition of Triton X-100 were established. To the remaining tubes, Mo-A was added to achieve final concentrations of 128, 64, 32, and 16 μg/mL. After incubation at 37 °C, the tubes were centrifuged, and the supernatant was collected. One hundred microliters of the supernatant were taken, and its optical density was measured at 600 nm. All measurements were performed in triplicate.

### In vivo toxicity test of Galleria mellonella

Final instar larvae of *Galleria mellonella* weighing between 220-260 mg were maintained under a controlled environment at 37 °C. They were randomly allocated into three groups, each comprising ten larvae: a control group treated with PBS and two treatment groups receiving 10, 25 or 50 mg/kg Mo-A. Administration of either PBS (containing 0.1% DMSO) for the control group or the drug was performed using a 10 μL Hamilton syringe, with the injection site being the last hole of the right leg of each larva. Over the subsequent five days, the survival status and degree of melanization of the larvae in all groups were assessed and documented.

### Hemolytic activity assay

The impact of Mo-A on the hemolytic activity of *S. aureus* USA300 and Newman was assessed by treating bacterial cultures with varying Mo-A concentrations at an initial OD_600_ of 0.3, followed by incubation until an OD_600_ of 2.5 was reached. In a 1 mL assay, 100 μL of culture supernatant, 25 μL of defibrinated rabbit blood, and 875 μL of PBS were combined. Negative controls included Triton X-100 and *S. aureus* supernatants, with other components remaining unchanged. After an hour incubation at 37 °C, supernatants were collected via centrifugation, and absorbance was measured at 543 nm. The supernatant of untreated bacterial cultures was used as a control for 100% hemolysis. The percent inhibition of hemolysis in the control culture was calculated. All measurements were performed in triplicate.

### qPCR

To elucidate the effect of Mo-A on the transcriptional activity of pivotal genes in *S. aureus*, we cultured the bacterium in the presence of variable Mo-A concentrations, initiating at an OD_600_ of 0.3 and extending into the log phase. Centrifugation was utilized to collect the bacteria, and total RNA was subsequently isolated utilizing TRIzol reagent, which was then reverse transcribed into cDNA. Quantitative real-time PCR (qRT‒PCR) was carried out employing qPCR SYBR Green premix (Vazyme, Nanjing, China) along with the primers delineated in Supplementary Table 1. The thermal cycling parameters encompassed an initial denaturation phase at 95 °C for 30 s followed by 40 cycles, each comprised of a denaturation step at 95 °C for 5 s, annealing at 60 °C for 30 s, and extension at 72 °C for 30 s. The obtained transcript levels were normalized to 16 S RNA and rplD which were evaluated through the 2-^ΔΔCT^ method. All measurements were performed in triplicate.

### Western blot

*S. aureus* cultures, initiated at an OD_600_ of 0.3, were subjected to different concentrations of Mo-A and further incubated until the late log phase. The bacteria were harvested, and total protein from each group was extracted using lysis buffer and quantified with a BCA Protein Assay Kit (Thermo Fisher Scientific). Subsequently, 40 μg of protein was separated by SDS‒PAGE and blotted onto a PVDF membrane. After sealing with 5% skim milk powder, the membrane was probed overnight at 4 °C with a 1:5000 dilution of anti-staphylococcal α-Toxin antibody produced in rabbit (cat# S7531-1ML, Sigma) or rabbit polyclonal to LukS - PVL (cat# ab190473, Abcam), and the secondary antibody was goat anti-rabbit IgG H&L (HRP) (cat# ab205718, Abcam). All measurements were performed in triplicate. After washing in Tris-buffered saline containing 0.1% Tween, the membrane was incubated for 1 h at room temperature with an HRP-conjugated goat anti-rabbit secondary antibody (1:5000 dilution). Membranes were visualized using an Enhanced Chemiluminescence Kit (Merck Millipore, Eschborn, Germany) and imaged with a chemiluminescence detection system. And the uncropped and unprocessed blot in Fig. 2 were shown in Fig. S5 in the Supplementary Information for illustrative purposes.

### Adherence of S. aureus to immobilized fibrinogen

To evaluate the adhesion capability of Mo-A-treated *S. aureus* to fibronectin, we initially coated 96-well plates with bovine FN solution (50 μg/mL, Source Leaf Company, Shanghai, China) and incubated them for 18 h. *S. aureus* without Mo-A treatment was the WT group, and the PBS group was the control group. Nonspecific adhesion was blocked by removing the FN solution and treating with 3% bovine serum albumin (Sigma Chemical Company) for 2 h. *S. aureus* was then grown to logarithmic phase in the presence of varying concentrations of Mo-A. Next, the solution in the 96-well plate was discarded and replaced with Mo-A-treated *S. aureus* (5 × 10^3^ CFU/well) and incubated at 37 °C for 1 h. After washing with phosphate-buffered saline (PBS), cells adhered to the plate were fixed with 4% formaldehyde for 30 min. The plate was washed twice with PBS and stained with crystal violet for 20 min, and the absorbance at 600 nm was measured using a microplate reader. All measurements were performed in triplicate.

### Analysis of nuclease activity

Supernatants derived from *S. aureus* cultures incubated in medium for 16–18 h were employed to quantify nuclease activity using a fluorescence resonance energy transfer (FRET) assay as previously described^55^. To ensure optimal detection within the linear range of the assay, these supernatants were diluted 100-fold with distilled water. Nuclease activity was denoted by the initial rate (Vinit) of the DNA cleavage reaction. V_init_ = Δ[S]/Δt, where V_init_ is the initial rate of the reaction, Δ[S] is the change in substrate concentration, and Δt is the change in time over the initial period. All measurements were performed in triplicate.

### Neutrophil isolation and chemotaxis assay

Peripheral blood from mice was collected, and neutrophils were isolated using a peripheral blood neutrophil isolation kit. These neutrophils were suspended in HBSS with 1% BSA. After incubating MRSA with 64 μg/mL Mo-A for 4 h at 37 °C and 220 rpm, the supernatant was collected. The MRSA was then heat-inactivated at 60 °C for 30 min. Neutrophil chemotaxis was assessed using a 24-well plate with 3.0 μm pore-sized transwell inserts. The inactivated MRSA (3 × 10^8^ cells/insert) was placed in the insert, and 0.6 ml HBSS with 1% BSA was placed in the lower chamber. Neutrophils were then exposed to the bacterial supernatant for 30 min at 37 °C, centrifuged, and suspended in HBSS with 1% BSA. Next, 1.5 × 10^4^ neutrophils were added to the upper wells and incubated for 1 h. After removing nonmigrated cells, neutrophils were stained with Ly6G (Alexa Fluor® 647), and after staining with the Ly6G-FITC antibody, the fluorescence intensity was measured at 535 nm with an excitation wavelength of 488 nm. All measurements were performed in triplicate.

### CETSA

CETSA exploits protein stability discrepancies under temperature fluctuations. Initially, bacterial lysates were partitioned, with one fraction treated with Mo-A (64 μg/mL) and another with DMSO as a control. Subsequently, mixtures are incubated at 37 °C for 1 h. After incubation, supernatants are procured via centrifugation and allocated to PCR tubes, followed by a 5-min incubation targeting MgrA protein at specific temperatures and a swift 3 min ice bath. Thereafter, samples were amalgamated with protein loading buffer and subjected to electrophoretic separation via 10% SDS‒PAGE. Ultimately, utilizing ImageJ software, quantitative analysis of band intensities correlated with protein stability was conducted to ascertain the impact of Mo-A on MgrA protein stability. All measurements were performed in triplicate.

### EMSA

EMSA was conducted according to a previously described method^37^. FAM-labeled DNA fragments containing promoter regions were synthesized as depicted in Supplementary Table 1. Binding reactions were established in reaction buffer with 10 mM Tris-HCl, pH 7.4, 50 mM KCl, 5 mM MgCl_2_, and 10% glycerol, comprising the MgrA protein, DNA probes, and Mo-A. Reactions were performed at 25 °C for 30 min. The samples were subjected to 5% native polyacrylamide gel electrophoresis. Visualization was conducted using a fluorescence gel imaging system, and grayscale analysis was performed with ImageJ software. All measurements were performed in triplicate.

### TSA

For the thermal shift assay with purified protein, 6×His-MgrA protein was purified and subsequently diluted in PBS to a final concentration of approximately 2 μM. The purified MgrA protein was then mixed with an equal volume of Sypro Orange (1:2000) and added to the TSA buffer system (10 mM Tris, 150 mM NaCl, pH = 7.5). Mo-A was then added to establish a 1:10 molar ratio with the MgrA protein, and the system was brought to a total volume of 20 μL with the addition of TSA buffer. Thermal scanning (25–90 °C, 1 °C/min) was carried out using a real-time PCR system (CFX384 - Bio-Rad Laboratories). All measurements were performed in triplicate.

### Fluorescence quenching

A fluorescence quenching assay was employed to quantify the binding constant (*K*_*A*_) between Mo-A and MgrA. The assay, comprising a total volume of approximately 1 mL, involved PBS buffer, purified MgrA protein (2 nM), and varying concentrations of Mo-A. The excitation wavelength was fixed at 280 nm with a 5-nm bandpass filter, and the emission slit width was set at 10 nm. The fluorescence emission spectrum, ranging from 290 to 450 nm, of the resulting solution was captured using a fluorescence spectrophotometer (RF5301, Japan). All measurements were performed in triplicate. Fluorescence quenching data were represented as the relative fluorescence intensity (RFI = F/F_0_ × 100) plotted against the Mo-A concentration, facilitating the construction of the Stern-Volmer plot of F_0_/F versus [Q]. The K_A_ values were derived through linear regression analysis. Detailed methodologies were previously reported^56^.

### Molecular modeling

A molecular docking study was performed to investigate the binding mode between Mo-A (PubChem: 74805-92-8, Compound CID: 53466984) and MgrA using AutoDock Vina 1.1.2^57^. The 3D structure of MgrA (PDB ID:2BV6) was downloaded from the Protein Data Bank (www.rcsb.org). The 3D structure of the compound was drawn by ChemBioDraw Ultra 14.0. The AutoDockTools 1.5.6 package^58,59^ was employed to generate the docking input files. Ligand structures were prepared for docking by merging nonpolar hydrogen atoms and defining rotatable bonds. The search grid of MgrA was identified as center_x: 72.053, center_y: 7.363, and center_z: 2.435 with dimensions size_x: 56.35, size_y: 56.35, and size_z: 56.35. To increase the docking accuracy, the exhaustiveness value was set to 20. For Vina docking, the default parameters were used if not mentioned.

### Site-directed mutagenesis design

Based on the binding sites obtained from the molecular docking simulation, we performed site-directed mutagenesis on the corresponding amino acid positions in the MgrA protein, changing them to alanine (Ala). If the original residue was already Ala, it was mutated to glycine (Gly) (The primers of mutagenesis in Supplementary Table 1). Subsequently, the mutated proteins were expressed and purified using standard induction methods. We further analyzed the inhibition rate of Mo-A on MgrA mutant proteins using fluorescence anisotropy and fluorescence quenching.

### Antibiotic combination

Sterile 96-well plates were arranged longitudinally, with the vertical axis representing the drug concentration of Mo-A and the horizontal axis representing the concentration of vancomycin. Mo-A was diluted continuously starting from a quarter of the MIC, while vancomycin was diluted continuously from the MIC. The *S. aureus* was then inoculated and cultured as described in the MIC. The potential synergistic effect is determined based on the FICI, which is calculated using the following formula:

(MIC combination)/(MIC alone)+(MIC antibiotic combination)/(MIC antibiotic alone)

If the FICI is ≤0.5, it indicates a synergistic effect; if it is >0.5 and ≤2.0, it indicates an additive effect; and if it is >2, it indicates an antagonistic effect.

### Bactericidal curve

A combination group (16 μg/mL of Mo-A + 1/4 MIC of vancomycin), a Mo-A group (16 μg/mL), a vancomycin group (1/4 MIC), and a blank control group were established. Each group was inoculated with USA300 to an initial bacterial concentration of 10^6^ CFU/mL. Subsequently, at 0 h, 4 h, 8 h, 12 h, and 24 h, 100 μL of bacterial solution was taken from each group, appropriately diluted, and then spotted on MHB solid medium. The cultures were continued until single colonies appeared for counting, and all measurements were performed in triplicate.

### Mo-A alone or in combination with vancomycin against MRSA-induced systemic infection in Galleria mellonella

*Galleria mellonella* for evaluating the therapeutic potential of Mo-A in a model of systemic infection with *S. aureus*. Five groups of larvae were established: the *S. aureus* USA300 infection group (WT), Mo-A treatment group, uninfected control group, vancomycin group, and combination group. Each group comprised 10 larvae (*n* = 10). The infection groups were inoculated with an MRSA bacterial suspension at a concentration of 5 × 10^8^ CFU/mL using a 10 μL volume. The inoculation was meticulously carried out at the distal region of the terminal left proleg^15^. One hour postinoculation, interventions commenced. The Mo-A treatment group received Mo-A doses of either 10 or 50 mg/kg, while the combination group was treated with both Mo-A (at the concentrations) and vancomycin at 50 mg/kg. The uninfected control group and the vancomycin group underwent their respective treatments, ensuring that no cross-treatments occurred. Throughout the experiment, larvae were maintained under controlled conditions, with ambient temperatures held consistently below 37 °C.

Over a span of 120 h, larval survival was meticulously recorded every 12 h. To gain a nuanced understanding of the health status of the larvae, a health index scoring system, delineated in Fig. 4D, was employed^60^. This system appraises the health of larvae by allocating scores grounded in four principal observations: larval mobility, cocoon formation, melanization, and overall survival.

The formula for calculating: total score per group = (Activity score + cocoon formation score + melanization score + survival score) × 10 (the number of each group)

### MRSA-induced mouse pneumonia model

Seven-week-old C57BL/6 J mice (approximately 22 grams) were obtained from Liaoning Changsheng Biotechnology Co., Ltd. (Changchun, China), provided ample water and food, and acclimated for one week. A mouse model of pneumonia induced by MRSA USA300 was established following previously reported literature^18^. Overnight cultures of *S. aureus* USA300 were subcultured into fresh TSB medium at a 1:100 ratio and incubated at 37 °C with shaking until log phase growth. The bacteria were collected by low-speed centrifugation and resuspended in PBS. The mice were randomly divided into five groups (*n* = 10): *S. aureus* USA300 infection group (WT), Mo-A treatment group (Mo-A), uninfected control group (control), vancomycin group (vancomycin) and vancomycin with Mo-A treatment group (combination). Each mouse was challenged with *S. aureus* USA300 (2 × 10^9^ CFU) for the survival study. The treatment group received a subcutaneous injection of 50 mg/kg Mo-A within one hour post *S. aureus* infection; the infection group was administered an equivalent volume of saline (0.05% DMSO) in the same manner. The vancomycin group received 50 mg/kg, while the combination group received 50 mg/kg Mo-A and 50 mg/kg vancomycin. Subsequent subcutaneous injections were administered every 12 h, and survival was monitored at 12 h intervals for up to 96 h^61,62^.

To assess bacterial burden and pulmonary pathological changes, mice were grouped (*n* = 8) and treated in a manner congruent with the survival analysis experiment. Mice were inoculated intranasally with USA300 (1 × 10^9^ CFU). Forty-eight hours postinfection, the mice were euthanized with 200 mg/kg euthanasia agent containing pentobarbital and cervical dislocation following the ARRIVE reporting guidelines^63^. And the right lung tissue was excised under sterile conditions, weighed, and homogenized. Following appropriate dilutions, the homogenate was plated onto TSA agar and incubated. Bacterial colonies were counted the following day. The left lung was photographed and then placed in formalin. After standard processes such as paraffin embedding and sectioning, the samples were further stained with H&E for observation under a light microscope.

In addition, cytokine concentrations in the bronchoalveolar lavage fluid of the mice (*n* = 6) were measured. The dosage and administration methods for the mice were identical to those employed in the bacterial burden analysis. Forty-eight hours posttreatment, 1 mL of sterile PBS was injected into the trachea, and the lavage fluid was collected for bronchoalveolar lavage fluid studies. The concentrations of cytokines (IL-6, cat#PI326; TNF-α, cat #PT512; IL-1β, cat #PI301, Beyotime, China) were evaluated using specific assay kits. These assays were meticulously carried out following the manufacturer’s protocols. Animal welfare and experimental protocols followed the ARRIVE guidelines. All procedures involving animals were approved by Animal Experimental Ethical Inspection of Changchun University of Chinese Medicine (Approval No. 2022171).

### Statistical analysis

Statistical evaluations of the data were conducted employing various analytical techniques, GraphPad Prism software (version 9.0) was utilized for all statistical analyses. Statistical evaluations of Figs. 1f–h, 2a–e, g–j, 3d, f, 5d, f-h, Supplementary Figs. S4, 6a and f, 9 were conducted employing analysis of variance (ANOVA) followed by Dunnett’s test. Statistical evaluations of Fig. 2f and Supplementary Figure 6b were conducted employing analysis of variance (ANOVA) followed by Tukey’s multiple comparisons test. Log rank tests were performed for survival analyses in Figs. 4b and 5c. *P* values are indicated in the figures. Statistical significance was inferred at a *P* value of ≤ 0.05. An asterisk (*) denotes *P* < 0.05, two asterisks (**) denote *P* < 0.01, and three asterisks (***) denote *P* < 0.001, n.s. denotes nonsignificant.

### Supplementary information

Supplementary Information for Thwarting Resistance: MgrA Inhibition with Methylophiopogonanone A Unveils a New Battlefront Against S. aureus

reporting-summary

## Data Availability

All data generated during this study are available upon request from the corresponding authors.
