# Supplementary material for: Thwarting resistance: MgrA inhibition with methylophiopogonanone a unveils a new battlefront against S. aureus
Source: NPJ Biofilms Microbiomes. 2024 Feb 27;10:15. doi: 10.1038/s41522-024-00485-w (PMC10899606; doi:10.1038/s41522-024-00485-w)
Supplement: Supplementary file 1 — Supplementary Information for Thwarting Resistance: MgrA Inhibition with Methylophiopogonanone A Unveils a New Battlefront Against S. aureus [file 41522_2024_485_MOESM1_ESM.pdf]

Supplementary Information for

Thwarting Resistance: MgrA Inhibition with Methylophiopogonanone A Unveils a New Battlefront Against *S. aureus*

Xuerui Guo[1,#], Li Wang[2,#], Jinlong Zhang[1,#], Quan Liu[3], Bingmei Wang[2], Da Liu[4], Fei Gao[5], Gongga Lanzi[6*], Yicheng Zhao[2,3,7*], Yan Shi[1*]

[1]School of Pharmaceutical Science, Jilin University, Changchun 130021, China
[2]Clinical Medical College, Changchun University of Chinese Medicine, Changchun 130117, China.
[3]Center for Pathogen Biology and Infectious Diseases, Key Laboratory of Organ Regeneration and Transplantation of the Ministry of Education, The First Hospital of Jilin University, Changchun, China
[4]School of Pharmacy, Changchun University of Chinese Medicine, Changchun, 130117, China
[5]Department of Laboratory Animals, College of Animal Sciences, Jilin University, Changchun, 130062, Jilin, China.
[6]Tibet University Medical College, Tibet, 850000, China
[7]State Key Laboratory for Diagnosis and Treatment of Severe Zoonotic Infectious Diseases, Key Laboratory for Zoonosis Research of the Ministry of Education, Institute of Zoonosis, and College of Veterinary Medicine, Jilin University, Changchun 130062, China.

# These authors contributed equally.
*Corresponding author: 35173588@qq.com (G. Lanzi), yichengzhao@live.cn (YC. Zhao), shiyan@jlu.edu.cn (Y. Shi)

This PDF file includes:

Supplementary Figure 1 to 9

Supplementary Table 1 to 4

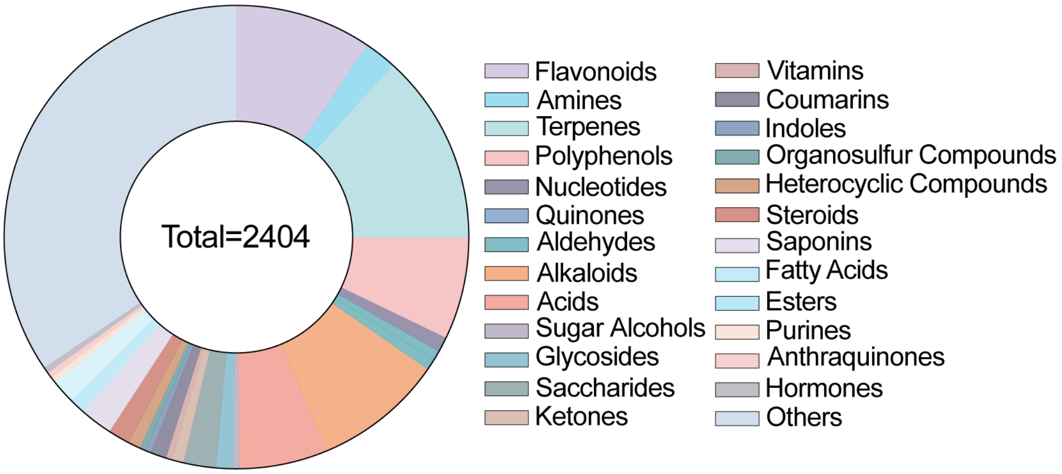

Supplementary Figure 1. Classification of compounds in the small molecule library for virtual screening.

The classification of all small molecules in the library used for virtual screening with Libdock was conducted. Molecules of the same type that numbered more than 10 were categorized into a distinct class, while others were grouped into the Others category, resulting in a total of 26 classifications.

Desite®
乐美天医药/德思特生物

基于标准 · 高于标准

Certificate of Analysis

甲基麦冬黄烷酮 A
Methylophiopogonanone A

CAS Number:74805-92-8
批号(Batch No.): DSTDM002201
分子式(M. F.):$C_{19}H_{18}O_6$
分子量(M. W.): 342.34

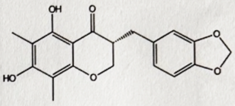

检验项目（ Test Item）	检验指标（ Specifications）	检验结果（ Results）
性状Appearance	Off-White powder	Conforms
纯度Purity (HPLC)	≥98.0%	99.72%
核磁NMR	Comply with the structure	Conforms
质谱Mass	Comply with the structure	Conforms

检测方法（Test Method）	Column:Ultimate XB-C18 4.6*250 mm,5μm; Column temperature: 35℃; Detection Mode: UV295nm; Flow Rate:1.0ml/min; Sample dissolution:Methanol; Mobile Phase:A-Acetonitrile ,B-0.1% Phosphoric acid in water;Gradient elution:A,57%,20min,57%-90%,5min.
贮存条件(Storage)	Keep Out of light, dry and stored at 2-8℃.
提示(Hint)	If you encounter quality problems, please contact us within 15 days after receiving the products and thank you for your purchase.
备注(Note)	The product is only used for scientific research experiments and should not be used for human injection, food or other purposes. This solution must be freshly prepared before using to avoid degradation of the purity of the product and affect the experimental results.

QC: chaoran zhong

QA: Chong chen

成都德思特生物技术有限公司
SpecialSeal:
质检章

Tel: 028-85003950
http://www.lemeitian.cn
Fax: 028-85589956
Add: 四川成都温江区海科路西段128号

Supplementary Figure 2. The qualification of Mo-A.

The purity of Mo-A is 99.72%.

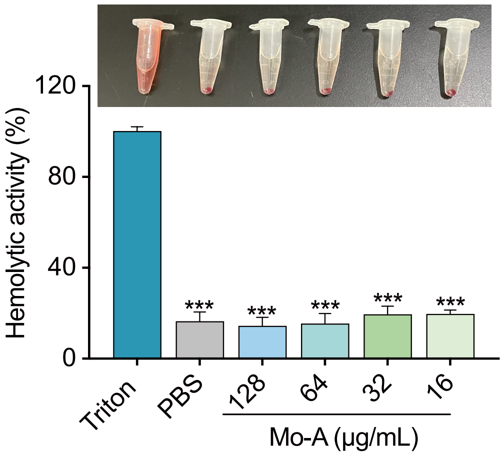

Supplementary Figure 3. The effects of different concentrations of Mo-A on the RBCs hemolytic activity.

Compared with triton group (Triton X-100 group), Mo-A did not produce hemolysis at different concentrations. Error bars represent standard error of the mean.

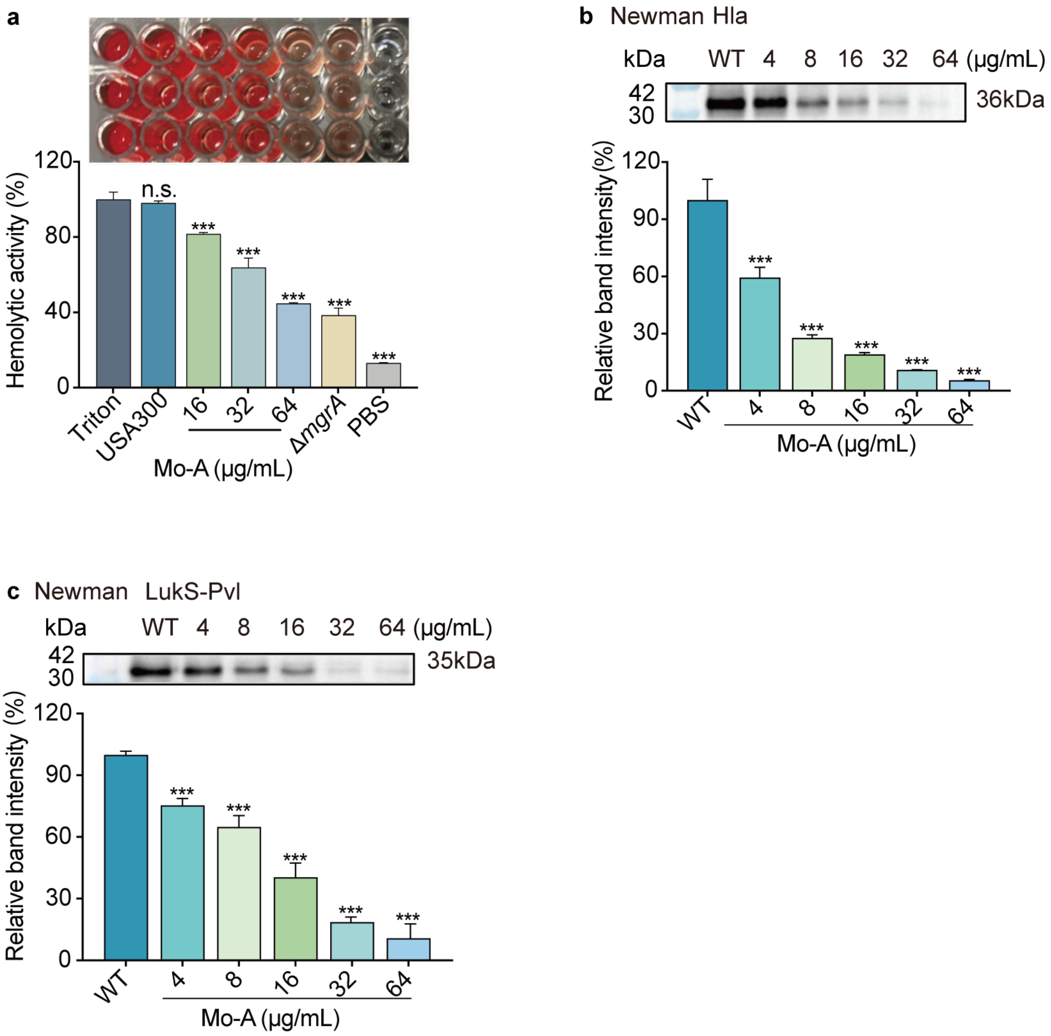

Supplementary Figure 4. The influence of Mo-A on the virulence factors regulated by MgrA.

(a) The effects of Mo-A on the hemolytic activity of *S. aureus* Newman. (b) Mo-A was found to inhibit the expression of Hla in *S. aureus* Newman. (c) Western blot analyses examined the impact of Mo-A on the expression of Pvl in *S. aureus* Newman. Error bars represent standard error of the mean. Immunoblot images have been cropped for illustrative purposes.

a

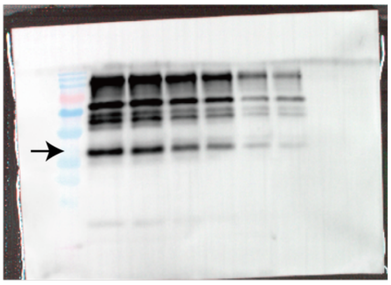

b

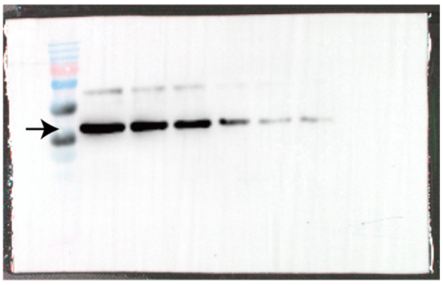

Supplementary Figure 5. Uncropped and unprocessed scans of western blot analyses examined the impact of Mo-A in *S. aureus* USA300.

Western blot analyses examined the impact of Mo-A on the expression of Hla (a) and Pvl (b) in *S. aureus* USA300.

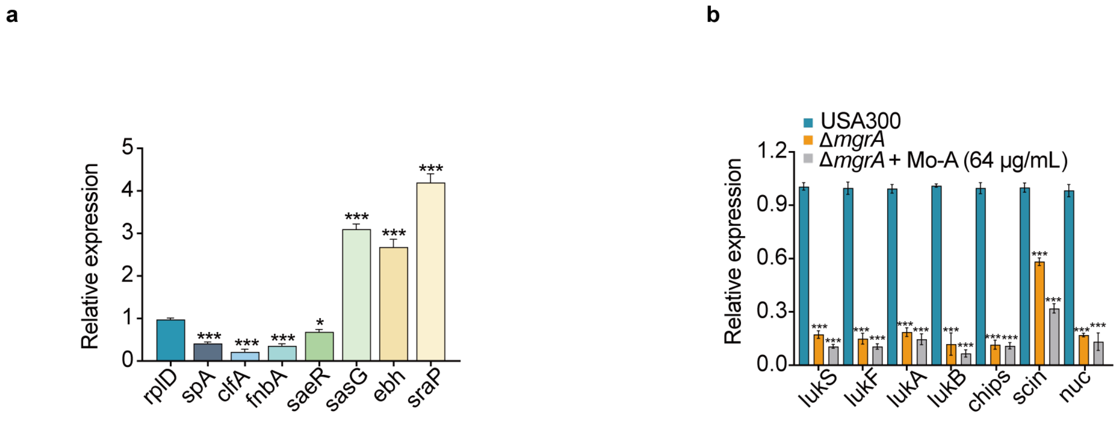

Supplementary Figure 6. Relative expression of immune evasion genes in *S. aureus*.

(a) Expression of immune evasion genes in mid-exponential *S. aureus* culture was measured with qPCR and normalized to rplD expression. Expression levels are relative to that of the USA300 strain, which is arbitrarily set at 1. (b) Expression of immune evasion genes in mid-exponential *S. aureus* culture was measured with qPCR and normalized to 16s RNA expression. Expression levels are relative to that of the USA300 strain, which is arbitrarily set at 1. Values are from three biological replicates. Error bars represent standard error of the mean.

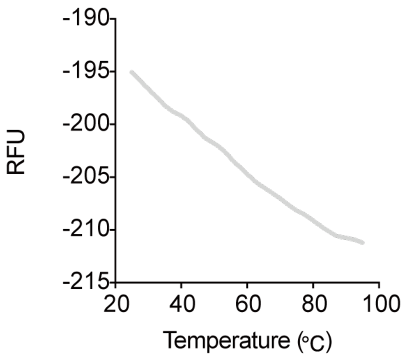

Supplementary Figure 7. Mo-A and fluorescence changes with increasing temperature.

Mo-A did not bind with the fluorophore with the increase of temperature from 25 to 100°C.

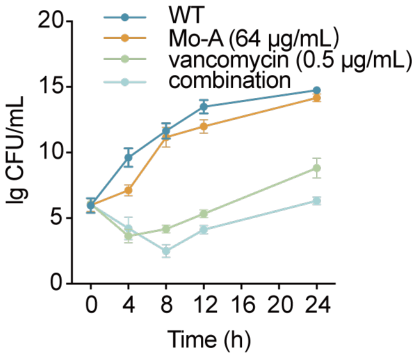

Supplementary Figure 8. Time-kill curve of Mo-A combined with vancomycin against USA300.

Compared to the WT group, Mo-A does not show a significant inhibitory effect on USA300. In the vancomycin group, the number of bacteria gradually decreased over time, reaching the lowest point at 12 h, showing a concentration-dependent effect. However, the combination of Mo-A and vancomycin significantly inhibited the growth of USA300. The number of bacteria reached the lowest at 12 h, and its antibacterial effect was significantly better than that of Mo-A or vancomycin used alone. Error bars represent standard error of the mean.

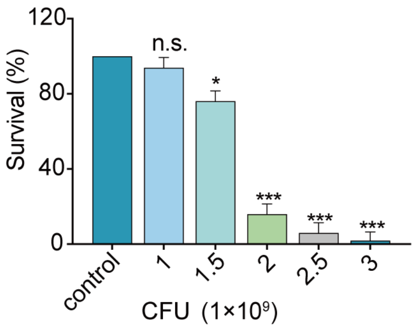

Supplementary Figure 9. The survival status of mice under different bacterial doses.

Compared to the control group without any bacterial inoculation, the changes in mice survival rates at different bacterial inoculation concentrations. Error bars represent standard error of the mean.

Supplementary Table 1. Primers used in this study.

Primers	Nucleotide sequence (5'→ 3')	Purpose
spA	TATGCCTAACTTAAATGCTG	qPCR
	GATGAAGCCGTTACGTTGTTC	
16S rRNA	CCATAAAGTTGTTCTCAGTT	qPCR
	CATGTCGATCTACGATTACT	
rplD	TTCGGACCAACTCCAAGA	qPCR
	CGAGCACCTCCTCAAC	
sraP	ACTGTAGGCAATCAAACCATAGA	qPCR
	CCGCTTGGTAATCCTGTAACT	
saeR	ATGAAAGGCTCTAAACAAATACT	qPCR
	ATCTTTTGTCATGAAATAAATGGG	
ebh	GTGAATTATCGTGATAAAATTCA	qPCR
	TTCCTACTGGCACTTGGTCTGC	
fnbA	ACTTGATTTTGTGTAGCCTTTTT	qPCR
	GAAGAAGCACCAAAAGCAGTA	
lukA	AGCTCAGGTGGTAAATTCGATTC	qPCR
	GACCAGTGTACATGCCAGTTATT	
lukB	GGACATGACCATACGAGACAAT	qPCR
	AACCCTTCAGACACAGTTACAG	
scin	AAATCTATACTTGCGGGAACTT	qPCR
	AAGCTTGTGCTAGCTTGTG	
lukS	CTGCAACATTGTCGTTAGGAATAA	qPCR
	CTCAGCGCCATCACCAATA	
lukF	GGCTTATCAGGTGGAGGTAATG	qPCR
	GCTTCAACATCCCAACCAATTT	
chips	CAGGAATCAGTACACACCATC	qPCR
	GCGTTGTAGGAAGACCACTATT	
nuc	CGAAAGGGCAATACGCAAAG	qPCR
	TGCATTTGCTGAGCTACTTAGA	
hla	GTGTACAAACGAAAAAGTATCGTATGTATTTTTAATATAG	EMSA
	TCCGGTACCTACGAGTTTCATTAACGTCACA	
Q19A	GAGTAGTAGCGATTAACTTGTCTTGCAGCATTGTACAAACTA AAGCATAG	PCR
	CTATGCTTTAGTTTGTACAATGCTGCAAGACAAGTTAATCGC TACTACTC	
Y38A	TAAGACAAGAAATTGTGGGGCTGTTAGATTGTACTTCTTAAA AACTTTGTTAGAGTAG	PCR
	CTACTCTAACAAAGTTTTTAAGAAGTACAATCTAACAGCCCC ACAATTTCTTGTCTTA	
N131A	ACTTTACCTAATAAGCGAGCAAGTTCTTTAACTTCATCTTGC GATAAAGAAGAAG	PCR
	CTTCTTCTTTATCGCAAGATGAAGTTAAAGAACTTGCTCGCT TATTAGGTAAAGT	

Supplementary Table 2. Molecular docking category

Name	Category	Types
A:GLN19- Structure2D_CID_53466984	Hydrogen Bond	Conventional Hydrogen Bond
A:TYR-38- Structure2D_CID_53466984	Hydrogen Bond	Conventional Hydrogen Bond
Structure2D_CID_53466984- A:SER113	Hydrogen Bond	Conventional Hydrogen Bond

Supplementary Table 3. Molecular docking score

Receptor	Ligand	Pubchem CID	Binging energy (kcal/mol)
MgrA	Mo-A	53466984	-6.7

Supplementary Table 4. The antibacterial effect of antibiotics combined with Mo-A against *S. aureus*

**Antibiotics**	**MIC** **(μg/mL)**	**FIC** **antibiotics**	**MIC $_{Mo-A}$** **(μg/mL)**	**FIC** **Mo-A**	**FICI**
Cefoxitin	8	0.5	512	0.125	0.625
Ceftriaxone Sodium	32	0.5	512	0.125	0.626
Cefaclor	8	1	512	0.125	1.125
Cefepime	128	1	512	0.125	1.125
Vancomycin	2	0.25	512	0.125	0.375
Ceftiofurna	16	2	512	0.125	2.125
Cefotaxime	16	1	512	0.125	1.125
Oxacillin	8	0.5	512	0.125	0.625
Penicillin G Sodium	512	0.5	512	0.125	0.625
Ceftazidime	32	2	512	0.125	2.125
